# Risk factors of persistent adolescent thinness: findings from the UK Millennium Cohort Study

**DOI:** 10.1186/s12889-023-15850-1

**Published:** 2023-05-24

**Authors:** H. Whitfield, D. Hargreaves, D. Nicholls, H. C. Watt, H. Creese

**Affiliations:** 1 School of Public Health, Imperial College London, London, UK; 2 Department of Brain Sciences, Imperial College London, London, UK

**Keywords:** Underweight adolescents, Thinness, Early-life characteristics, Psychological characteristics, Genetic determinants, Body mass index

## Abstract

**Background:**

Thinness during adolescence can increase the risk of adverse health outcomes across the life-course and impede development. There is limited research examining the prevalence and determinants of persistent adolescent thinness in the United Kingdom (UK). We used longitudinal cohort data to investigate determinants of persistent adolescent thinness.

**Methods:**

We analyzed data from 7,740 participants in the UK Millennium Cohort Study at ages 9 months, 7, 11, 14 and 17 years. Persistent thinness was defined as thinness at ages 11, 14 and 17; thinness was defined as an age- and sex-adjusted Body Mass Index (BMI) of less than 18.5 kg/m^2^. In total, 4,036 participants, classified either as persistently thin or at a persistent healthy weight, were included in the analyses. Logistic regression analyses were conducted to examine associations between 16 risk factors and persistent adolescent thinness by sex.

**Results:**

The prevalence of persistent thinness among adolescents was 3.1% (*n* = 231). Among males (*n* = 115), persistent adolescent thinness was significantly associated with non-white ethnicity, low parental BMI, low birthweight, low breastfeeding duration, unintended pregnancy, and low maternal education. Among females (*n* = 116), persistent adolescent thinness was significantly associated with non-white ethnicity, low birthweight, low self-esteem, and low physical activity. However, after adjusting for all risk factors, only low maternal BMI (OR: 3.44; 95% CI:1.13, 10.5), low paternal BMI (OR: 22.2; 95% CI: 2.35, 209.6), unintended pregnancy (OR: 2.49; 95% CI: 1.11, 5.57) and low self-esteem (OR: 6.57; 95% CI: 1.46,29.7) remained significantly associated with persistent adolescent thinness among males. After adjustment for all risk factors, not reaching the recommended physical activity levels (OR: 4.22; 95% CI: 1.82, 9.75) remained significantly associated with persistent adolescent thinness among females. No appreciable associations were found between persistent adolescent thinness and sex, premature birth, smoking during pregnancy, income, maternal postnatal depression, mother-infant attachment or socio-emotional difficulties (*p* > 0.05).

**Conclusion:**

Persistent adolescent thinness is not rare and appears to be associated with both physical and mental health factors, with some sex specific differences. Healthy weight initiatives should consider the full weight spectrum. Further research is required to understand thinness at a population level, including among those whose BMI changes during child and adolescent development.

**Supplementary Information:**

The online version contains supplementary material available at 10.1186/s12889-023-15850-1.

## Background

Adolescent thinness, as defined as an equivalent Body Mass Index (BMI) of less than 18.5 kg/m^2^ at 18 years old adjusted for age and sex (BMI Standard Deviation score = -1) [1], can have serious consequences on both health and development. Specifically, adolescent thinness is associated with stunted growth, delayed maturation, nutritional deficiencies and reduced cognitive capability [2]. Furthermore, thinness can increase the risk of osteoporosis, a weakened immune system, anaemia, respiratory failure, wound complications, extended hospital stays, infertility in women, preterm birth and an increased risk of all-cause mortality [3–6]. In the United Kingdom (UK), it is estimated that between 5 to 15% of the population accessing healthcare services are classified as underweight [5]. Investigating thinness during adolescence is of value, as adolescence is a period of rapid growth with adequate nutrition being crucial to achieve full growth potential, peak bone mass and optimal organ development [3]. Furthermore, adolescence is associated with increased autonomy and independence, including laying the foundations for lifelong decisions concerning diet and physical activity [7].

There are a variety of factors which could determine an individual’s risk of thinness including biological, early-life, socio-economic, psychological and life-style factors. Risk factors for persistent thinness are likely different to risk factors for thinness status lost or gained. Regarding biological factors, certain genetic loci have been found to be associated with an increased risk of thinness [8], as well as certain ethnicities, specifically people with a South Asian background [9]. Certain medical conditions can result in persistent adolescent thinness; chronic conditions including Inflammatory Bowel Disease are commonly diagnosed during adolescence [10]. Early-life factors, including a low birthweight and prematurity, have also been shown to be associated with childhood and adolescent thinness [11]. A previous UK study found that children from more disadvantaged socio-economic groups were more likely to be underweight as well as obese [12]; this may be a reflection of inadequate nutrition and food poverty which is more prevalent among disadvantaged households in the UK [13]. Furthermore, children from low socio-economic status households are also less likely to participate in sports [14], often due to a lack of opportunities, and are therefore less likely to build up adequate muscle mass. Persistent thinness can also be a result of certain mental disorders, including Avoidant/Restrictive Food Intake Disorder (ARFID). ARFID commonly presents before the age of 12 and in some cases can be characterized by a persistent low BMI and an inability to achieve appropriate weight gain [15, 16]. Parental influence, including parental support, parenting styles and attachment, also can have a large impact on a child’s eating behavior and BMI [17].

The rationale for investigating persistent thinness, as opposed to thinness incidence, is that longer duration of thinness is associated with an increased risk of adverse health [18, 19]. Furthermore, since puberty is associated with rapid changes in physical structure, body composition and biological maturation this can influence puberty-dependent incidental low BMI [20]. Persistent thinness is therefore a more robust measure of adolescent thinness than thinness at a single time point.

The aim of this study is to examine associations between biological, early-life, socioeconomic, psychological, and life-style factors and persistent adolescent thinness among both males and females using data from the UK Millennium Cohort Study (MCS).

## Methods

### Study design & population

Data for this study were obtained from the Millennium Cohort Study (MCS), a longitudinal study of children born in the United Kingdom (UK) between September 2000 and January 2002. Participating families were randomly sampled from electoral wards, with a stratified cluster sampling design to safeguard representation of all four UK countries, disadvantaged and ethnically diverse areas. Survey interviews were carried out in the home of the main respondent which in 99.8% of cases was the biological mother at 9 months. We used data from sweeps when children were 9 months, 7, 11 14, and 17 years old.

There were 18,296 singleton children included in the first MCS data collection sweep at age 9 months. By the age of 17 years 7,740 singleton children had participated in all waves. Of these cohort members, 7,721 had complete records regards Body Mass Index (BMI) at ages 11, 14 and 17. Our study focused on the 4,036 adolescents who had a BMI classified as either persistently underweight or persistently healthy weight. Those who did not fall into either of these categories (*n* = 3,685) were omitted from the analysis as they were not the focus of this study.

### Study variables

#### Persistent adolescent thinness

Sex- and age-specific International Obesity Task Force (IOTF) BMI cut-offs were used to classify adolescents as underweight, healthy weight, overweight or obese [1]. Persistent thinness was defined as those who were classified as underweight at ages 11, 14 and 17. BMI, defined as weight in kilograms over height in meters squared, was calculated using height and weight measurements taken by the interviewer using a standardized protocol [21].

#### Risk factor variables 

We identified potential risk factors for adolescent thinness through a scoping literature review and matched them to available variables in the MCS (Table 1).

**Table 1:** Summary of risk factors of adolescent thinness

Risk Factor*		**Categories**	**Data Wave**	**Reported by**
**Biological**	Sex	Male	9 months	Main respondent
		Female		
	Ethnicity	White	9 months	Main respondent
		Indian		
		Pakistani & Bangladeshi		
		Black		
		Other		
	Maternal BMI	Underweight (< 18.5 BMI)	9 months	Biological mother
		Healthy weight (≥ 18.5 & < 25.0 BMI)		
		Overweight/Obese (≥ 25.0 BMI)		
	Paternal BMI	Underweight (< 18.5 BMI)	9 months	Biological father
		Healthy weight (≥ 18.5 & < 25.0 BMI)		
		Overweight/Obese (≥ 25.0 BMI)		
**Early-life**	Birthweight	Low birthweight (< 2500 g)	9 months	Main respondent
		Healthy birthweight (≥ 2500 g & < 4000 g)		
		High birthweight (≥ 4000 g)		
	Premature Birth	Premature (Gestational age < 37 weeks)	9 months	Main respondent
		Not Premature (Gestational age ≥ 37 weeks)		
	Smoking During Pregnancy	Smoked	9 months	Biological Mother
		Did not smoke		
	Breastfeeding	Breastfed for over 3 months	9 months	Main respondent
		Breastfed less than 3 months		
	Planned Pregnancy	Planning to get pregnant	9 months	Main respondent
		Pregnancy was a surprise		
**Socio-economic**	Maternal Education	No educational qualification	9 months	Main respondent
		NVQ Level 1		
		NVQ Level 2		
		NVQ Level 3		
		NVQ Level 4		
		NVQ Level 5		
	Income Quintile	Lower quintile	9 months	Main respondent
		Second quintile		
		Third quintile		
		Fourth quintile		
		Highest quintile		
**Psychological**	Maternal Postnatal Depression	Postnatal Depression (< 14 Rutter Malaise Score)	9 months	Biological Mother
		No postnatal depression (≥ 14 Rutter Malaise Score)		
	Mother-Infant Attachment	Secure Attachment (< 21 Condon Attachment Score)	9 months	Biological Mother
		Insecure Attachment (≥ 21 Condon Attachment Score)		
	Socio-emotional Difficulties	Low (0–13 SDQ Score)	7 years	Cohort Member’s Parents
		Borderline/High (17–40 SDQ Score)		
	Self-esteem	Low self-esteem (> 11 Rosenberg Score)	11 years	Cohort Member
		Healthy self-esteem (≤ 11 Rosenberg Score)		
**Life-Style**	Physical Activity	Met Recommended Physical Activity Levels (≥ 60 min activity per day)	7, 11, 14 and 17 years	Main respondent (ages 7 and 11) and cohort member (age 14 and 17)
		Below Recommended Physical Activity Levels (< 60 min activity per day)		

^*^Risk factors were identified through a scoping literature review

##### Biological

At the first sweep when the child was 9 months old, the main respondent reported the child’s sex and ethnicity. Biological maternal and paternal BMI was only available at the 9-month sweep; parents’ current height and weight was self-reported, and these data were used to categorize parents into underweight, healthy weight and overweight/obese categories according to IOTF BMI cut-offs [1].

##### Early-life

The main respondent also reported the child’s birthweight which was categorized according to classifications set out by the UK World Health Organization [22]. Prematurity was defined as a gestational age of less than 37 weeks; gestational age was reported by the main respondent at the 9-month interview. Smoking during pregnancy was self-reported by the natural mother. Breastfeeding was self-reported by the main respondent and the child was defined as having been breastfed if they had been given any breast milk for 3 calendar months. Whether or not the pregnancy was planned was also reported by the main respondent.

##### Socioeconomic

The main respondent also reported their highest level of academic or vocational education attained at the 9-month interview which was then classified into six National Vocational Qualification (NVQ) categories. Family income was self-reported by the main respondent at the 9-month interview and was then divided into quintiles.

##### Psychological

Maternal postnatal depression was assessed for biological mothers at the 9-month interview using nine questions from the Rutter Malaise Inventory [23]. Mothers who reported scores in the lowest 10^th^ percentile (below a score of 14) were classified as having postnatal depression. Mother-infant attachment quality was assessed when the child was 9 months old using six questions from the Condon Maternal Postnatal Attachment Scale [24]. Adolescents whose mother reported an attachment score in the lowest 10^th^ percentile (below a score of 21) were classified as having poor attachment. To measure the child’s mental health, the Strengths and Difficulties Questionnaire (SDQ) was given to the child’s parents at the 7-year interview. SDQ scores were then divided into low difficulties (0–13) and borderline/high difficulties (14–40) [25]. Self-esteem was assessed using items from the Rosenberg scale when the cohort member was 11-years old for the first time, and those who reported scores in the highest 10^th^ percentile (above a score of 11) were classified as having a low self-esteem.

##### Life-style

Physical activity data were reported at ages 7, 11, 14 and 17. A binary longitudinal physical activity variable was created of those who reached UK National Health Service (NHS) recommended physical activity levels, of at least 60 min of moderate intensity physical activity a day, across all ages compared to those who did not [26].

### Statistical analysis

Three different models of adjustment were used to analyze the association between risk factors and adolescent thinness; an unadjusted model, model adjusted for ethnicity, and a mutually adjusted model. To examine the univariate associations between biological, early-life, socioeconomic, psychological, and life-style factors, and persistent adolescent thinness, chi-squared tested were used. The model, adjusted for ethnicity, used binomial logistic regression to estimate the odds ratios (OR) for the association between risk factor variables and persistent adolescent thinness by sex. The mutually adjusted model adjusted for all predictor factors which were found to be statistically associated with persistent adolescent thinness in either sex, as determined by a *p* value < 0.05. The data used in this analysis included one row per cohort member.

#### Sensitivity analyses

To validate the results of the mutually adjusted model, a mixed effects logistic regression model was performed for all predictor factors which were found to be statistically associated with persistent adolescent thinness in either sex, as determined by a *p* value < 0.05. Adolescent age was used as the random intercept. The data used for the mixed effects logistic regression model included three rows per cohort member; age 11, 14 and 17.

Attrition and response weight were applied to the analyses, using *svy* commands in Stata, to account for the stratified clustering sample design and loss to follow-up [27]. All statistical analyses were conducted in Stata version 15.1 (StataCorp, College Station, TX, USA).

### Ethics, consent, and permissions

The Millennium Cohort Study was approved by the Southwest and London Multi-Centre Research Ethics Committees, has been fully anonymized. Additional ethics review was not required for this secondary analysis.

## Results

### Prevalence of persistent thinness

Of the 7,721 cohort members for whom information regarding Body Mass Index (BMI) was available at 11-, 14- and 17-years-old, 231 (3.1%) adolescents were found to be persistently underweight at the three time points. Furthermore, 3,805 (49.3%) were found to have been at a persistently healthy weight, 458 (5.9%) were found to be persistently overweight, 238 (3.1%) were persistently classified as obese and 2,989 (38.7%) were found to fluctuate between BMI categories between the ages of 11 and 17 (Table 2). While persistently underweight adolescents accounted for 3.1% of the total cohort, after omitting cohort members who were persistently overweight, obese or moved between BMI categories during adolescence, persistently underweight adolescents accounted for 5.7% (*n* = 231) of the analytical sample (*n* = 4,036).

**Table 2:** Number of adolescent participants by persistent BMI category in the Millennium Cohort Study, UK 2000–2018

**BMI Category**	**Number**	**Percentage**
Persistently Underweight	231	3.1%
Persistently Healthy Weight	3805	49.3%
Persistently Overweight	458	5.9%
Persistently Obese	238	3.1%
BMI category changed during adolescence	2989	38.7%

BMI categories are determined by International Obesity Task Force cut-offs according to age and gender

BMI was measured at ages 11, 14 and 17

As shown in Table 3, the prevalence of persistent adolescent thinness varied by risk factor variable. Adolescent thinness was found to be more prevalent among adolescents from non-white ethnicities, adolescents with underweight parents, adolescents who were born with a low birthweight, adolescents whose mother had no or NVQ Level 1 education, adolescents whose mothers suffered from postnatal depression, adolescents with low self-esteem, and adolescents who had not met the recommended physical activity levels throughout adolescence. When, the prevalence of persistent adolescent thinness was examined by sex, adolescent thinness was more prevalent among female adolescents from non-white ethnicities, among females with low self-esteem and among females who have not reached the recommended physical activity levels throughout adolescence. Conversely, adolescent thinness was more prevalent among male adolescents with underweight parents, males whose mother had no or NVQ Level 1 education, and males whose mothers suffered from postnatal depression.

**Table 3:** Association between risk factor variables and adolescent weight in the Millennium Cohort Study, UK 2000–2018

**Cohort Member Characteristics**		**n**	**Persistent Healthy Weight**	**Persistently Underweight**	***p*** **-value**
**Sex**	Male	4036	53.1% (1,928)	53.0% (115)	0.990
	Female		46.9% (1,877)	47.0% (116)	
**Ethnicity**	White	4029	85.8% (3,237)	74.1% (154)	0.003
	South Asian		7.2% (339)	15.1% (57)	
	Black & Other		7.0% (222)	10.8% (20)	
**Maternal BMI**	Underweight	3563	4.59% (124)	13.6% (27)	< 0.001
	Healthy weight		65.7% (2,209)	71.3% (140)	
	Overweight/Obese		29.7% (1,032)	15.1% (31)	
**Paternal BMI**	Underweight	3,264	0.4% (8)	4.1% (3)	< 0.001
	Healthy Weight		44.0% (701)	54.3% (53)	
	Overweight/Obese		55.6% (875)	41.6% (31)	
**Birthweight**	Low birthweight	4036	5.46% (198)	13.8% (34)	< 0.001
	Healthy birthweight		82.4% (3,135)	80.4% (182)	
	High birthweight		12.2% (472)	5.81% (15)	
**Premature Birth**	Premature	3999	6.17% (228)	8.93% (22)	0.194
	Not Premature		93.8% (3,541)	91.1% (208)	
**Smoking During Pregnancy**	Smoked	4035	14.5% (435)	13.7% (22)	0.824
	Did not smoke		85.6% (3,359)	86.3% (209)	
**Breastfeeding**	Breastfed for over 3 months	4030	27.8% (1,145)	22.9% (64)	0.179
	Breastfed less than 3 months		72.2% (2,654)	77.1% (167)	
**Planned Pregnancy**	Planning to get pregnant	2366	53.7% (1,301)	46.0% (73)	0.127
	Pregnancy was a surprise		46.3% (928)	54.0% (64)	
**Maternal Education**	No educational qualification	3929	13.9% (383)	19.4% (34)	0.026
	NVQ Level 1		7.58% (219)	13.3% (22)	
	NVQ Level 2		28.4% (919)	23.7% (50)	
	NVQ Level 3		14.8% (568)	11.1% (29)	
	NVQ Level 4 & 5		35.4% (1,624)	32.5% (81)	
**Income Quintile**	Lower quintile	4024	18.3% (564)	25.5% (51)	0.142
	Second quintile		19.6% (691)	21.5% (50)	
	Third quintile		18.6% (700)	14.0% (31)	
	Fourth quintile		20.9% (862)	17.6% (47)	
	Highest quintile		22.6% (978)	21.4% (50)	
**Maternal Postnatal Depression**	Postnatal Depression	3901	13.0% (430)	18.4% (38)	0.044
	No postnatal depression		87.0% (3,252)	81.6% (181)	
**Mother-Infant Attachment**	Secure Attachment	3401	87.8% (2,822)	84.8% (152)	0.290
	Insecure Attachment		12.3% (397)	15.2% (30)	
**Socio-emotional Difficulties**	Healthy	3724	88.8% (3,204)	86.8% (175)	0.498
	Borderline abnormal		11.2% (318)	13.2% (27)	
**Self-esteem**	Low self-esteem	3731	9.19% (288)	15.9% (24)	0.015
	Healthy self-esteem		90.8% (3,236)	84.1% (183)	
**Physical Activity**	Met Recommended Physical Activity Levels	3802	58.6% (2,181)	45.9% (96)	0.004
	Below Recommended Physical Activity Levels		41.3% (1,410)	54.1% (115)	

Values are given as Percentage (Number)

### Model adjusted for ethnicity

After adjusting for ethnicity, logistic regression analysis showed that among males; South Asian ethnicity compared to white (OR: 2.47; 95% CI:1.45,4.21), having a mother with a BMI classified as underweight compared to healthy weight (OR: 3.54; 95% CI:1.70,7.39), having a father with a BMI classified as underweight compared to healthy weight (OR:8.35; 95% CI:1.67,41.9) a low birthweight compared to healthy weight (OR: 2.37; 95% CI:1.11,5.06), being breastfed for less than 3 months compared to being breastfed for longer than 3 months (OR: 1.75; 95% CI:1.03,3.03), being born as a result of an unplanned pregnancy compared to a planned pregnancy (OR: 1.77; 95% CI:1.04,3.00), having a mother with NVQ Level 1 education compared to NVQ Level 4 and 5 (OR: 2.58; 95% CI:1.28, 5.20), and being in the lowest income quintile compared to the highest (OR: 2.13, 95% CI:1.04,4.39) were associated with an increased odds of persistent adolescent thinness. Among females, South Asian ethnicity compared to white (OR: 2.38; 95% CI:1.41,4.03), a low birthweight compared to a healthy birthweight (OR: 2.49; 95% CI:1.22,5.08), low self-esteem compared to healthy self-esteem (OR: 2.03; 95% CI:1.03,3.99) and not reaching the recommended physical activity levels compared to reaching the recommended levels (OR: 1.76; 95% CI:1.05,2.97) were associated with an increased odds of persistent adolescent thinness (Table 4).

**Table 4:** Odds ratios obtained by logistic regression analysis adjusted for ethnicity, Millennium Cohort Study, UK 2000–2018

**Risk Factor Variables**		**Sample Size**	**Persistent Adolescent Thinness- Males**	**Persistent Adolescent Thinness—Females**
			**OR (95% CI)**	**OR (95% CI)**
**Sex**	Male	4029	1	
	Female		0.99 (0.74, 1.35)	
**Ethnicity** ^**a**^	White	4029	1	1
	South Asian		2.47 (1.45,4.21)**	2.38 (1.41,4.03)**
	Black & Other		1.80 (0.58, 5.56)	1.76 (0.74,4.15)
**Maternal BMI**	Healthy Weight	3557	1	1
	Underweight		3.54 (1.70, 7.39)**	1.01 (0.34, 2.95)
	Overweight/Obese		0.43 (0.21, 0.87)*	0.50 (0.27, 0.95)*
**Paternal BMI**	Healthy Weight	3257	1	1
	Underweight		8.35 (1.67, 41.9)*	0.45 (0.06, 3.28)
	Overweight/Obese		0.63 (0.37, 1.07)	0.29 (0.17, 0.49)***
**Birthweight**	Healthy birthweight	4029	1	1
	Low birthweight		2.37 (1.11, 5.06)*	2.49 (1.22, 5.08)*
	High birthweight		0.49 (0.24, 1.01)	0.56 (0.17, 1.81)
**Premature Birth**	Not Premature	3992	1	1
	Premature		1.68 (0.75, 3.77)	1.26 (0.63, 2.55)
**Smoking During Pregnancy**	Did not smoke	4028	1	1
	Smoked		1.05 (0.55, 2.00)	1.06 (0.39, 2.84)
**Breastfeeding**	Breastfed less than 3 months	4023	1	1
	Breastfed for over 3 months		0.57 (0.33, 0.97)*	1.04 (0.61, 1.78)
**Planned Pregnancy**	Planning to get pregnant	2362	1	1
	Pregnancy was a surprise		1.77 (1.04, 3.00)*	0.86 (0.47, 1.55)
**Maternal Education**	NVQ Level 4 & 5	3922	1	1
	NVQ Level 3		0.76 (0.33,1.77)	0.81 (0.43, 1.50)
	NVQ Level 2		1.22 (0.69,2.15)	0.69 (0.38,1.24)
	NVQ Level 1		2.58 (1.28, 5.20)**	1.41 (0.49,4.10)
	No educational qualification		1.98 (0.96, 4.07)	0.68 (0.34, 1.36)
**Income Quintile**	Highest quintile	4017	1	1
	Fourth quintile		0.79 (0.37, 1.69)	0.95 (0.52, 1.76)
	Third quintile		0.86 (0.39, 1.90)	0.71 (0.31, 1.65)
	Second quintile		1.31 (0.63, 2.75)	0.77 (0.40, 1.53)
	Lowest quintile		2.13 (1.04, 4.39)*	0.63 (0.34, 1.25)
**Maternal Postnatal Depression**	No Postnatal Depression	3894	1	1
	Postnatal Depression		1.71 (0.92, 3.17)	1.14 (0.59, 2.18)
**Mother-Infant Attachment**	Secure Attachment	3394	1	1
	Insecure Attachment		0.96 (0.49, 1.89)	1.61 (0.82, 3.17)
**Socio-emotional Difficulties**	Healthy	3717	1	1
	Borderline abnormal		1.09 (0.53, 2.23)	0.43 (0.16, 1.15)
**Self-esteem**	Healthy self-esteem	3724	1	1
	Low self-esteem		1.91 (0.93, 3.93)	2.03 (1.03, 3.99)*
**Physical Activity**	Met Recommended Physical Activity Levels	3795	1	1
	Below Recommended Physical Activity Levels		1.40 (0.85, 2.30)	1.76 (1.05, 2.97)*

The logistic regression model, adjusted for ethnicity, shows the odds ratio of persistent adolescent thinness compared with persistent healthy weight among participants at ages 11,14 and 17

*OR* Odds ratio

*CI* Confidence Interval

^*^ *p* < 0.05, **p < 0.01, ***p < 0.001

^a^Ethnicity results are unadjusted for any other variables

### Mutually adjusted model

Risk factors which were associated with persistent adolescent thinness after adjusting for ethnicity in either sex were included in a multivariable regression model, where 1,271 cohort members had information regarding all variables (Table 5). Among males, having a mother with a BMI classified as underweight (OR: 3.44; 95% CI:1.13, 10.5), having a father with a BMI classified as underweight (OR: 22.2; 95% CI:2.35,209.6), unintended pregnancy (OR: 2.49; CI:1.11,5.57) and low self-esteem (OR: 6.57; 95% CI:1.46, 29.7) were predictive of persistent adolescent thinness. Among females, not reaching the recommended physical activity levels (OR: 4.22; 95% CI: 1.82, 9.75) was predictive of persistent of adolescent thinness, and having a father with a BMI classified as overweight/obese (OR:0.20; 95% CI:0.07,0.56), and having a mother with no educational qualification (OR: 0.05; 95% CI: 0.00, 0.65) was found to be a protective factor against persistent adolescent thinness. The mixed effects logistic regression sensitivity analysis confirmed the association between parental BMI, maternal education, self-esteem and physical activity found in the multivariable logistic regression model (Additional file 1). The sensitivity analysis also found an association between ethnicity, income and adolescent thinness in boys, and maternal BMI, birthweight, breastfeeding, planned pregnancy, income, self-esteem and adolescent thinness in girls.

**Table 5:** Multivariable logistic regression model showing the odds ratios, Millennium Cohort Study, UK 2000–2018 (*n* = 1,271)

**Cohort Member Characteristics**		**Persistent Adolescent Thinness**	
		**Males** **OR (95% CI)**	**Females** **OR (95% CI)**
**Ethnicity**	White	1	1
	South Asian	1.26 (0.32, 4.94)	1.25 (0.26, 5.98)
	Black & Other	4.69 (0.95, 23.1)	0.55 (0.09, 3.32)
**Maternal BMI**	Healthy weight	1	1
	Underweight	3.44 (1.13, 10.5)*	1.57 (0.32, 7.66)
	Overweight/Obese	0.38 (0.14, 1.06)	0.56 (0.15, 2.13)
**Paternal BMI** ^**a**^	Healthy Weight	1	1
	Underweight	22.2 (2.35, 209.6)**	-
	Overweight/Obese	0.73 (0.32, 1.67)	0.20 (0.07, 0.56)**
**Birthweight**	Healthy birthweight	1	1
	Low birthweight	0.31 (0.07, 1.41)	4.13 (0.77, 22.0)
	High birthweight	0.75(0.19, 2.95)	0.82 (0.14, 4.72)
**Breastfeeding**	Breastfed less than 3 months	1	1
	Breastfed for over 3 months	0.91 (0.41, 2.03)	1.36 (0.53, 3.52)
**Planned Pregnancy**	Planning to get pregnant	1	1
	Pregnancy was a surprise	2.49 (1.11, 5.57)*	0.46 (0.14, 1.56)
**Maternal Education**	NVQ Level 4 & 5	1	1
	NVQ Level 3	1.26 (0.38, 4.13)	0.19 (0.02, 0.53)
	NVQ Level 2	1.30 (0.50, 3.38)	0.48(0.14, 1.60)
	NVQ Level 1	1.11 (0.19, 6.37)	0.31 (0.04, 2.22)
	No educational qualification	1.34 (0.29, 6.26)	0.05 (0.00, 065)*
**Income**	Highest quintile	1	1
	Fourth quintile	0.96 (0.25, 3.67)	4.09 (0.95, 17.5)
	Third quintile	0.61 (0.16, 2.39)	4.63 (0.95, 22.6)
	Second quintile	0.60 (0.13, 2.84)	0.33 (0.05, 2.29)
	Lowest quintile	3.25 (1.06, 9.91)	11.4 (0.90, 142.7)
**Self-esteem**	Healthy self-esteem	1	1
	Low self-esteem	6.57 (1.46, 29.7)*	3.02 (0.81, 11.3)
**Physical Activity**	Met Recommended Physical Activity Levels	1	1
	Below Recommended Physical Activity Levels	1.13 (0.52, 2.72)	4.22 (1.82, 9.75)**

The multivariable logistic regression model, mutually adjusted for all risk factors, shows the odds ratio of persistent adolescent thinness compared with persistent healthy weight among participants at ages 11, 14 and 17

*OR* Odds ratio

*CI* Confidence Interval

^*^ *p* < 0.05, **p < 0.01, ***p < 0.001

^a^Underweight Paternal BMI predicts failure perfectly for persistent thinness in adolescent females, and has therefore been omitted

## Discussion

We found that 3.1% of UK adolescents during the period of 2012 to 2018 were persistently underweight as defined by the International Obesity Task Force (IOTF) cut off, consistent with previous studies examining the prevalence of adolescent thinness [28, 29]. The proportion of persistently underweight adolescents was found to be the same (3.1%) as that of persistently obese adolescents in this sample. This is converse to previous findings which have found obesity to be more prevalent than thinness among adolescents [30]. Nonetheless, the Millennium Cohort Study (MCS) is a representative population cohort, suggesting that thinness prevalence may be worth exploring in other samples. However, persistent weight status throughout adolescence likely has different determinants and manifestations compared to a single BMI measurement. Our findings suggest that more adolescents move in and out of being obese, compared with thinness. This could be explained by the prevalence of obesity increasing as age increases, as well as the presence of several policies and strategies present to tackle obesity in children and adolescents [30, 31]. Comparatively there are very few policies tackling thinness.

We found that among males, having an underweight mother, underweight father, resulting from an unintended pregnancy and low self-esteem all increased the risk of being persistently underweight across the ages of ages of 11, 14 and 17. Among females we found that those who did not reach recommended physical activity levels had higher odds of persistent thinness across the ages of 11, 14 and 17. The sensitivity analysis, using mixed effects models to account for the longitudinal data, provided additional support that these predictor variables are associated with adolescent thinness. We found little evidence (*p* > 0.05) that any of the following predict adolescent thinness: sex, premature birth, smoking during pregnancy, maternal postnatal depression, mother-infant attachment or socio-emotional difficulties as measured by the total SDQ score.

All Body Mass Index (BMI) categories have been found to be inter-generationally correlated, and while it has been suggested that this could be due to both shared environment and genetics, BMI has only been found to be significantly associated between biological parent–child pairs, suggesting that inter-generational BMI transference occurs primarily through genetic mechanisms [32]. This suggestion is supported by the findings of genetic loci which are associated with obesity and thinness [8]. This finding also concurs with studies investigating genetic risk for anorexia nervosa, which found a negative correlation with high BMI, leading to suggestions that both risk for thinness and risk for mental disorder may be implicated in the etiology [33].

We found an association between low self-esteem at age 11 and persistent adolescent thinness among males. While there is strong evidence to suggest that low self-esteem is critical in fueling disordered eating [34], adolescents with eating disorders are likely to only make up a small proportion of persistently underweight male adolescents [35]. The pathway between low self-esteem and thinness could be mediated by negative affect, including low mood or anxiety, similar to the pathway between low self-esteem and eating disorders. However, we did not find a role for total Strengths and Difficulties Questionnaire (SDQ) score in predicting adolescent thinness. Alternatively, persistent thinness could conversely be a cause of low self-esteem in males, as it is with obesity [36]. Future analyses could consider more specific mental health risk factors such as depression and anxiety.

Female adolescents who did not consistently exercise at recommended levels from the age of 7, were shown to have an increased odd of persistent adolescent thinness. Whereas previous studies have not used longitudinal measures of exercise, lower physical activity levels have still been found to be associated with thinness during adolescence [37]. There are a number of potential explanations for the association between lower physical activity levels and thinness. A lack of adequate exercise can result in lower muscle mass among adolescents, which in turn can lower BMI due to the relative mass of muscle compared to other components of body composition [38]. Food insecurity and malnutrition can cause significant health problems, which can in turn lead to persistent fatigue and therefore a reduced capability to partake in physical exercise [39]. Furthermore, long-term conditions which are associated with thinness including musculoskeletal conditions, epilepsy, and mental health conditions can impair an individual’s ability to perform physical exercise [40–42].

Previous studies have found there to be a strong association between maternal education and weight status, with a low maternal education being associated with an increased risk of childhood adiposity in high income countries [43–45]. Since children and adolescents with mothers with lower educational qualifications have a higher risk of being overweight, this could also be acting as a protective factor against persistent adolescent thinness. This suggests that persistent adolescent thinness may not be as a result of a lack of health-related knowledge, but rather reflect wider socio-economic factors. Furthermore, high parental education has been identified as a risk factor for eating disorders [46]. Therefore, it is possible as a result of encouragement for the child to become a high achiever and a desire for perfectionism that this could result in persistent adolescent thinness.

We did not find a significant association between early-life factors and adolescent thinness. This suggests that while early-life factors are important in determining childhood weight, as children develop into adolescents, other factors become more relevant.

### Strengths and limitations

The strengths of our study include the use of data from a large, representative UK cohort, which follows the cohort members from birth to adolescence, and also the wide range of risk factors which were analyzed in determining the risk of adolescent thinness. To our knowledge, this study is the first to conduct a descriptive epidemiological review concerning the determinants of persistent thinness in both male and female adolescents.

The MCS is a highly comprehensive cohort study, with multiple time points of data collection and an exhaustive variable list. This allowed us to include biological, early-life, socio-economic, psychological, and life-style risk factors in our analysis. However, some additional risk factors, such as diet, have not been comprehensively captured in the MCS.

Furthermore, we used an objective measurement of adolescent thinness. The IOTF BMI cut-offs are one of the most widely used methods of identifying thinness, accounting for sex and age [1]. However, BMI does not consider body fat, muscle mass, or bone density and therefore is considered an acceptable yet imperfect measure of thinness.

Many of the variables, including maternal BMI, in the MCS are self-reported and therefore susceptible to information bias. Despite the size of the MCS, statistical power was limited when cohort members were broken down by ethnicity. Therefore, we pooled data across ethnic minorities and we suggest that more research be performed on datasets containing greater representation from ethnic minorities.

As with all prospective cohort studies, the MCS suffers from attrition and missing data. At baseline, the MCS compromised of 18,296 singleton children, however, only 7,740 singleton adolescents participated in all waves up to the age of 17. To minimize attrition bias, we used response weights to account for the loss of respondents up to 17 years. The attrition weights adjust the sample composition to take account of the selective loss of respondents, for example, low-income families who may be less likely to remain in the cohort. More information on the MCS attrition weights can be found on the Centre for Longitudinal Studies website [47].

Of the adolescents still participating at age 17 years, 7,721 (99.7%) had complete BMI records of which 4,036 were classified as persistently underweight healthy or persistently at a healthy weight. Depending on the variable (Tables 3 and 4), univariate analyses were performed on between 2,362 and 4,036 adolescents, and the multivariate analysis included 1,271 adolescents with complete data. Whilst using the MCS allowed for the investigation of a wide range of risk factors of persistent thinness, their inclusion considerably reduced the final sample size. Adolescents with missing data are more likely to be from disadvantaged socio-economic backgrounds, which may underestimate the association between certain socio-economic factors and persistent thinness.

#### Policy implications

To our knowledge, this is the first longitudinal study to examine the determinants of persistent adolescent thinness in a representative UK sample. Thinness, relative to obesity, receives little attention from policymakers. This study provides an opportunity to highlight the prevalence and determinants of adolescent thinness, which has detrimental health impacts. Policymakers should consider both ends of the BMI spectrum when tackling healthy weight among children and adolescents. Our data suggest comparable complexity in terms of genetic, sociodemographic and environmental as well as psychological risk factors playing a role in adolescent thinness, as they do for obesity. The role of health inequalities, including poverty and belonging to a minority ethnic group, need to be better understood and addressed. More research is needed to investigate the long-term outcomes of adolescent thinness and the efficacy of early interventions to mitigate negative outcomes. Since many of the predictors of adolescent thinness, including maternal BMI and a lack of physical exercise, are similar to predictors of obesity, current obesity policies and interventions, such as the National Child Measurement Programme, could feasibly be extended to target children and adolescents who are underweight and might go some way toward redressing the weight bias for which they have been criticized [48, 49].

## Conclusion 

Thinness during adolescence has been shown to be a risk factor for adverse health outcomes later in life, yet the UK has very few approaches regarding prevention. This study has shown that adolescent thinness is associated with low maternal BMI, low self-esteem, insufficient physical activity and maternal education. Therefore, these factors provide important areas for intervention in order to combat adolescent thinness. Further research is needed to understand those who move in and out of thinness during adolescence to understand risk factors and examine whether they differ from those with persistent thinness.

## Supplementary Information

**Additional file 1: Table S1.** Mixed effects logistic regression model showing the odds ratios, Millennium Cohort Study, UK 2000-2018 (*n*=2,678).

## Data Availability

The datasets analysed during the current study are available through the UK Data Service, https://beta.ukdataservice.ac.uk/datacatalogue/series/series?id=2000031

## Abbreviations

ARFID: Avoidant/Restrictive Food Intake Disorder
BMI: Body Mass Index
CI: Confidence Interval
IOTF: International Obesity Task Force
MCS: Millennium Cohort Study
NHS: National Health Service
NVQ: National Vocational Qualification
OR: Odds Ratio
SDQ: Strengths and Difficulties Questionnaire
UK: United Kingdom

## References

[1] Cole TJ, Lobstein T (2012). Extended international (IOTF) body mass index cut-offs for thinness, overweight and obesity. Pediatr Obes.

[2] Schönbeck Y, Van Dommelen P, HiraSing RA, Van Buuren S (2015). Thinness in the era of obesity: trends in children and adolescents in the Netherlands since 1980. Eur J Public Health.

[3] Das JK, Salam RA, Thornburg KL, Prentice AM, Campisi S, Lassi ZS (2017). Nutrition in adolescents: physiology, metabolism, and nutritional needs. Ann N Y Acad Sci.

[4] Dobner J, Kaser S (2018). Body mass index and the risk of infection - from underweight to obesity. Clin Microbiol Infect.

[5] Elia M (2015). The cost of malnutrition in England and potential cost savings from nutritional interventions.

[6] Worldwide trends in body-mass index (2017). underweight, overweight, and obesity from 1975 to 2016: a pooled analysis of 2416 population-based measurement studies in 128·9 million children, adolescents, and adults. Lancet.

[7] Soenens B, Vansteenkiste  M, Van Petegem S (2017). Autonomy in Adolescent Development. Autonomy in Adolescent Development.

[8] Riveros-McKay F, Mistry V, Bounds R, Hendricks A, Keogh JM, Thomas H (2019). Genetic architecture of human thinness compared to severe obesity. PLoS Genet.

[9] Toftemo I, Jenum AK, Lagerløv P, Júlíusson PB, Falk RS, Sletner L (2018). Contrasting patterns of overweight and thinness among preschool children of different ethnic groups in Norway, and relations with maternal and early life factors. BMC Public Health.

[10] Baldassano RN, Piccoli DA (1999). Inflammatory Bowel Disease in Pediatric and Adolescent Patients. Gastroenterol Clin North Am.

[11] Goyal NK, Fiks AG, Lorch SA (2012). Persistence of underweight status among late preterm infants. Arch Pediatr Adolesc Med.

[12] Pearce A, Rougeaux E, Law C (2015). Disadvantaged children at greater relative risk of thinness (as well as obesity): a secondary data analysis of the England National Child Measurement Programme and the UK Millennium Cohort Study. Int J Equity Health.

[13] Miller BDD, Welch RM (2013). Food system strategies for preventing micronutrient malnutrition. Food Policy.

[14] Sport England (2019). Active Lives Children and Young People Survey.

[15] American Psychiatric Association (2013). Diagnostic and Statistical Manual of Mental Disorders..

[16] Fisher MM, Rosen DS, Ornstein RM, Mammel KA, Katzman DK, Rome ES (2014). Characteristics of Avoidant/Restrictive Food Intake Disorder in Children and Adolescents: A “New Disorder” in DSM-5. J Adolesc Health.

[17] Savage JS, Fisher JO, Birch LL (2007). Parental influence on eating behavior: Conception to adolescence. Journal of Law, Medicine and Ethics. NIH Public Access.

[18] Piñar-Gutiérrez A, Dios-Fuentes E, Remón-Ruiz P, del Can-Sánchez D, Vázquez-Morejón A, López-Narbona M (2021). Description of characteristics and outcomes of a cohort of patients with severe and enduring eating disorders (SE-ED). J Eat Disord.

[19] Fonville L, Giampietro V, Williams SCR, Simmons A, Tchanturia K (2014). Alterations in brain structure in adults with anorexia nervosa and the impact of illness duration. Psychol Med.

[20] Albertsson-Wikland K, Niklasson A, Gelander L, Holmgren A, Nierop AFM (2022). Novel type of references for BMI aligned for onset of puberty – using the QEPS growth model. BMC Pediatr.

[21] Fitzsimons E, Agalioti-Sgompou V, Calderwood L, Gilbert E, Haselden L, Johnson J (2017). Millennium Cohort Study Sixth Survey 2015–2016 User Guide.

[22] RCPCH-WHO baby growth charts for 0–4 years. https://www.rcpch.ac.uk/resources/uk-who-growth-charts-0-4-years. Accessed 18 Sep 2021.

[23] Rutter M, Tizard J, Whitmore K (1970). Education, Health And Behaviour.

[24] Condon JT, Corkindale CJ (1998). The assessment of parent-to-infant attachment: development of a self-report questionnaire instrument. J Reprod Infant Psychol.

[25] Goodman R (1997). The Strengths and Difficulties Questionnaire: a research note. J Child Psychol Psychiatry.

[26] Physical activity guidelines for children and young people - NHS. https://www.nhs.uk/live-well/exercise/physical-activity-guidelines-children-and-young-people/. Accessed 16 Jul 2020.

[27] Mostafa T, Ploubidis G (2015). Millennium Cohort Study.

[28] Taylor SJC, Viner R, Booy R, Head J, Tate H, Brentnall SL (2005). Ethnicity, Socio-economic Status, Overweight and Underweight in East London Adolescents. Ethn Health.

[29] O’Dea JA, Chiang H, Peralta LR (2014). Socioeconomic patterns of overweight, obesity but not thinness persist from childhood to adolescence in a 6 -year longitudinal cohort of Australian schoolchildren from 2007 to 2012. BMC Public Health.

[30] Baker C (2022). Obesity statistics.

[31] NHS. The NHS long term plan. 2019. https://www.longtermplan.nhs.uk/.

[32] Classen TJ, Thompson O (2016). Genes and the intergenerational transmission of BMI and obesity. Econ Hum Biol.

[33] Watson H, Yilmaz Z, Thornton L, Hübel C, Coleman J, Gaspar H (2019). Genome-wide association study identifies eight risk loci and implicates metabo-psychiatric origins for anorexia nervosa. Nat Genet.

[34] Zelkowitz RL, Cole DA (2019). Self-Criticism as a Transdiagnostic Process in Nonsuicidal Self-Injury and Disordered Eating: Systematic Review and Meta-Analysis. Suicide Life Threat Behav.

[35] Smink FRE, Van Hoeken D, Hoek HW (2012). Epidemiology of eating disorders: Incidence, prevalence and mortality rates. Curr Psychiatry Rep.

[36] Sagar R, Gupta T (2018). Psychological Aspects of Obesity in Children and Adolescents. Indian J Pediatr.

[37] Kantanista A, Osiński W (2014). Underweight in 14 to 16 year-old girls and boys: prevalence and associations with physical activity and sedentary activities.

[38] McCarthy HD, Samani-Radia D, Jebb SA, Prentice AM (2014). Skeletal muscle mass reference curves for children and adolescents. Pediatr Obes.

[39] Lukaski H (2004). Vitamin and mineral status: effects on physical performance. Nutrition.

[40] Bettany-Saltikov J, Parent E, Romano M, Villagrasa M, Negrini S (2014). Physiotherapeutic scoliosis-specific exercises for adolescents with idiopathic scoliosis. Eur J Phys Rehabil Med.

[41] Arida R, Scorza F, Terra V, Scorza C, de Almeida A, Cavalheiro E (2009). Physical exercise in epilepsy: what kind of stressor is it?. Epilepsy Behav.

[42] Zhao G, Ford E, Dhingra S, Li C, Strine T, Mokdad A (2009). Depression and anxiety among US adults: associations with body mass index. Int J Obes (Lond).

[43] Feng Y, Ding L, Tang X, Wang Y, Zhou C (2019). Association between maternal education and school-age children weight status: a study from the China health nutrition survey, 2011. Int J Environ Res Public Health.

[44] Ruiz M, Goldblatt P, Morrison J, Porta D, Forastiere F, Hryhorczuk D (2016). Impact of Low Maternal Education on Early Childhood Overweight and Obesity in Europe. Paediatr Perinat Epidemiol.

[45] Lê-Scherban F, Moore J, Headen I, Utidjian L, Zhao Y, Forrest CB (2021). Are there birth cohort effects in disparities in child obesity by maternal education?. Int J Obes (Lond).

[46] O’Brien KM, Whelan DR, Sandler DP, Hall JE, Weinberg CR (2017). Predictors and long-term health outcomes of eating disorders. PLoS One.

[47] CLS | Millennium Cohort Study. https://cls.ucl.ac.uk/cls-studies/millennium-cohort-study/. Accessed 31 Jul 2022.

[48] Nnyanzi L, Summerbell C, Ells L, Shucksmith J (2016). Parental response to a letter reporting child overweight measured as part of a routine national programme in England: results from interviews with parents. BMC Public Health.

[49] Mairs R, Nicholls D (2016). Assessment and treatment of eating disorders in children and adolescents. Arch Dis Child.

