# Supplementary material for: Risk factors of persistent adolescent thinness: findings from the UK Millennium Cohort Study
Source: BMC Public Health. 2023 May 24;23:938. doi: 10.1186/s12889-023-15850-1 (PMC10207649; doi:10.1186/s12889-023-15850-1)
Supplement: Supplementary file 1 — Additional file 1: Table S1. Mixed effects logistic regression model showing the odds ratios, Millennium Cohort Study, UK 2000-2018 (n=2,678). [file 12889_2023_15850_MOESM1_ESM.docx]

**Additional file 1**

**Table S1: Mixed Effects Logistic Regression Model Showing the Odds Ratios, Millennium Cohort Study, UK 2000-2018 (n=2,678).**

| Cohort Member Characteristics | | Adolescent Thinness | |
| --- | --- | --- | --- |
|  |  | Males  OR (95% CI) | Females  OR (95% CI) |
| Ethnicity | White | 1 | 1 |
|  | South Asian | 1.52 (0.70, 3.32) | 2.32 (0.98, 5.50) |
|  | Black & Other | 2.41 (1.09, 5.33)* | 1.61 (0.66, 3.90) |
| Maternal BMI | Healthy weight | 1 | 1 |
|  | Underweight | 2.94 (1.50, 5.75)** | 3.36 (1.35, 8.33)** |
|  | Overweight/Obese | 0.43 (0.24, 0.75)** | 0.49 (0.27, 0.88)* |
| Paternal BMI^1^ | Healthy Weight | 1 | 1 |
|  | Underweight | 8.92 (1.34, 59.3)* | - |
|  | Overweight/Obese | 0.67(0.43, 1.05) | 0.23 (0.14, 0.38)*** |
| Birthweight | Healthy birthweight | 1 | 1 |
|  | Low birthweight | 1.49 (0.58, 3.82) | 8.29 (3.94, 17.4)*** |
|  | High birthweight | 1.08 (0.56, 2.06) | 0.70 (0.28, 1.72) |
| Breastfeeding | Breastfed less than 3 months | 1 | 1 |
|  | Breastfed for over 3 months | 0.82 (0.51, 1.32) | 2.02 (1.24, 3.28)** |
| Planned Pregnancy | Planning to get pregnant | 1 | 1 |
|  | Pregnancy was a surprise | 1.35 (0.85, 2.15) | 0.45 (0.25, 0.81)** |
| Maternal Education | NVQ Level 4 & 5 | 1 | 1 |
|  | NVQ Level 3 | 1.14 (0.60, 2.18) | 0.31 (0.14, 0.67)** |
|  | NVQ Level 2 | 1.32 (0.73, 2.39) | 0.75 (0.42, 1.37) |
|  | NVQ Level 1 | 1.61 (0.66, 3.91) | 0.50 (0.13, 1.96) |
|  | No educational qualification | 1.67 (0.68, 4.10) | 0.07 (0.01, 0.33)** |
| Income | Highest quintile | 1 | 1 |
|  | Fourth quintile | 1.40 (0.78, 2.50) | 3.38 (1.83, 6,25)*** |
|  | Third quintile | 0.48 (0.22, 1.08) | 4.36 (2.17, 8.77)*** |
|  | Second quintile | 0.62 (0.28, 1,39) | 0.72 (0.26, 2.01) |
|  | Lowest quintile | 3.45 (1.62, 7.32)** | 7.02 (2.35, 20.9)*** |
| Self-esteem | Healthy self-esteem | 1 | 1 |
|  | Low self-esteem | 2.51 (1.15, 5.50)* | 2.07 (1.03, 4.16)* |
| Physical Activity | Met Recommended Physical Activity Levels | 1 | 1 |
|  | Below Recommended Physical Activity Levels | 1.46 (0.92, 2.31) | 4.41 (2.69, 7.20)*** |

OR= Odds ratio.

CI = Confidence Interval.

* p<0.05, **p<0.01, ***p<0.001

^1^ Underweight Paternal BMI predicts failure perfectly for persistent thinness in adolescent females, and has therefore been omitted.
